# Supplementary material for: Effect of nanoencapsulation on the solubility and antioxidant activity of astaxanthin pigmented oil extracted from shrimp waste (Litopeneaus vannamei)
Source: PLoS One. 2024 Nov 15;19(11):e0313059. doi: 10.1371/journal.pone.0313059 (PMC11567521; doi:10.1371/journal.pone.0313059)
Supplement: Attachment — Submitted filename: Response to Reviewers - round 1.docx [file pone.0313059.s002.docx]

**RESPONSE TO REVIEWERS**

Reviewer #1:- The manuscript is based on the nanoencapsulation of astaxanthin with a concluding remark for usage as a food matrix

*R. Thank you for considering our manuscript. We hope the revised manuscript is suitable for publication in Plos One.*

- If that being the finding, preliminary study for human consumption needs to be checked and that's a future prospect.

*R. We appreciate the comment. The initial objective of this work was to evaluate the encapsulation of pigmented oil using porcine gelatin and soy protein, characterizing these nanoparticles and evaluating some bioactive properties. As a next step of the research, toxicity tests will be performed to ensure the safety of the particles.*

- In the materials and methods many parameters are studied, shell life of the nanoencapsulation needs to be discussed

*R. This study evaluated whether the wall materials efficiently promoted the nanoencapsulation of astaxanthin-pigmented oil, and the effects of nanoencapsulation on the bioactive potential of buriti oil. The nanoparticles have not yet been added to the food matrix, as further studies are needed to achieve their application. Therefore, shelf-life tests will be performed in the future.*

- If administered orally how does the absorption take place in the gut of the animal?

*R. The investigation of digestion and absorption in vitro or in vivo was not the objective of this study. First, different wall materials were evaluated to find the most efficient one to promote the nanoencapsulation of the astaxanthin-pigmented oil through the O/W emulsification technique, to provide the oil with solubility in an aqueous matrix and preserve or enhance the antioxidant activity. Based on this study, in the future, the nanoparticles will be evaluated for toxicity and bioactive potential in vitro and in vivo, aiming to elucidate other questions and expand the knowledge about the nanoparticles produced. It is expected that the digestion of the nanoparticles will occur in the stomach, since protein encapsulating agents were used, with controlled release of the buriti oil containing the bioactive compounds.*

- The images Fig.3, Fig.4, Fig.5, Fig.6 needs to have higher resolution.

*R. We appreciate the comments and provide a better view of Figures 3, 4. 5 and 6.*

Reviewer #2

A detailed study and analysis of the hypothesis and laboratory work have been observed. All the required characterzation and instrumenation have been utilized, and the required data have been presented in a scientific and readable manner.

*R. Thank you for considering our manuscript. We hope the revised manuscript is suitable for publication in Plos One.*
